# Using qualitative approaches to improve quantitative inferences in environmental psychology

**DOI:** 10.1016/j.mex.2020.100943

**Published:** 2020-05-28

**Authors:** Neil A. Lewis, Mario Bravo, Sarah Naiman, Adam R. Pearson, Rainer Romero-Canyas, Jonathon P. Schuldt, Hwanseok Song

**Affiliations:** a Cornell University; b Environmental Defense Fund; c Pomona College; d Purdue University

**Keywords:** Community-based research, Mixed methods, Focus groups

## Abstract

This article describes the qualitative approach used to generate and interpret the quantitative study reported by Song and colleagues’ (2020) in their article, “What counts as an ‘environmental’ issue? Differences in environmental issue conceptualization across race, ethnicity, and socioeconomic status.” Song and colleagues (2020) describe the results of a survey documenting that, in the United States, White and high-SES respondents perceive environmental issues differently than their non-White and lower-SES counterparts, reflecting structural differences in environmental risks. While Song and colleagues (2020) discuss the survey results in detail, the discussion of the qualitative research that led to the creation of that survey was limited due to space constraints. The current article provides a more holistic account of the methods behind the Song and colleagues (2020) study by discussing the qualitative component of the research in detail. In addition to discussing how the qualitative research complements and critically informs the findings reported by Song et al., we also consider the broader implications and value of integrating qualitative and quantitative methods in environmental psychology.

• Conduct qualitative study to inform quantitative design.
• Use qualitative patterns to make inferences about quantitative indicators.

## Graphical abstract

**Figure:**
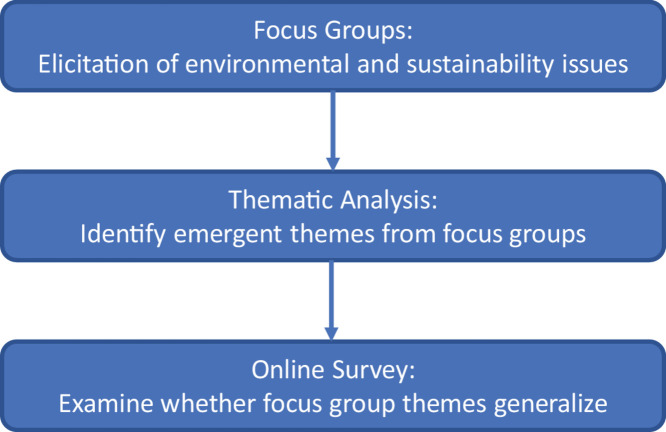

Specifications table

**Table:** 

Subject Area	Psychology
More specific subject area	*Social Psychology*
Method name	*Mixed methods in environmental psychology*
Name and reference of original method	*N/A*
Resource availability	*N/A*

## Background and study rationale

Research in social psychology has consistently documented that people's embodied traits and characteristics, social relationships, roles, and group memberships shape not only how we come to define ourselves [28], but also how we perceive the world around us, and act on those perceptions. That is, our social contexts and identities provide a lens through which to interpret and make meaning of the world around us [20,29]. They also allow us to determine which groups we belong to [42]. And, because social institutions make group differences salient through differential treatment [34], group memberships end up shaping how we process and make sense of our experiences [19], and understand the differences between our experiences and the experiences of others with different attributes [36]. This finding—that our social contexts and identities shape perception, cognition, and action has been well-document across a variety of domains (c.f., [30]), including environmental psychology.

Research over the past few decades increasingly documents differences in the ways that individuals think about environmental issues as a function of their race, ethnicity, and socioeconomic status (SES) group membership, variables that serve as proxies for the differential experiences that people have in segregated and otherwise stratified social contexts [33,^35^,^37^,45]. Take, for instance, the sizable literature on racial/ethnic differences in environmental attitudes. Numerous studies reveal that—contrary to prevailing stereotypes—racial/ethnic minorities express comparable and oftentimes greater levels of environmental concern and elevated environmental risk perceptions, relative to Whites (e.g., [13,^14^,^17^,^25^,41]). At the same time, work in public opinion finds that Americans substantially underestimate the environmental concerns of non-Whites and lower-income respondents —an “environmental belief paradox” observed even among non-White and lower-income respondents [31]. That is, in the United States for example, nationally representative probability sample studies have documented that the groups that are *most* concerned about the environment are Latina/os, Low-income Americans, Asian Americans, and African Americans (in that order), but those are the very groups that Americans misperceive to be the *least* concerned about the environment [31]. Although different explanations for these misperceptions have been offered, a common account involves the assumption that these groups have more immediate concerns, such as concerns about employment, that preclude them from prioritizing the environment (for a review, see [12]).

Research on environmental justice (see [26]) and environmental racism [33,45] offers an alternative perspective on group differences in environmental concern. In particular, the environmental deprivation hypothesis posits that heightened levels of environmental concern expressed by minority and lower-SES communities reflect awareness of the disproportionate environmental risks (e.g., pollution exposure; [45]) these communities face, as well as recognition of social conditions that can exacerbate these risks [33]. Supporting this reasoning, a substantial literature documents that minorities and lower-SES groups are exposed to relatively greater levels of environmental hazards (e.g., air and water pollution) [24,48].

Moreover, minority and lower-income communities face a “double jeopardy” when it comes to environmental threats – not only are they often more exposed to environmental hazards, but, due to systemic social inequities, they are also more sensitive to that exposure, particularly in urban areas [38,47]. For instance, low-income housing sites are often more exposed to environmental hazards, and poor infrastructure, and limited access to transportation and other public services can further amplify social vulnerability, and reduce the capacity of communities to mobilize to address environmental problems (for reviews, see [10]; and [38]).

Whereas early risk research on environmental hazards emphasized vulnerability due to the hazard itself (e.g., flooding due to coastal erosion, the nature and composition of pollutants), more recent work on “social vulnerability” has focused on the underlying social conditions, including systemic political and economic inequality, segregation, structural racism, unequal access to transportation, limited opportunities for education, weak infrastructure, and existing health disparities, that make humans vulnerable – the focus of environmental justice [6] and environmental racism research [45]. Neighborhood-level factors, including access to transportation, healthy food, and critical services, including healthcare, parks, open spaces, social environments, crime rates, and physical features of urban environments (e.g., traffic density, housing quality) can further shape vulnerability to environmental hazards [23].

Additional work has revealed the ways in which environmental hazards, in turn, can expose and exacerbate existing social inequities, as illustrated by the disproportionate effects of Hurricane Katrina on minority and low-income communities in the U.S. Gulf Coast [8]. A sizable literature on vulnerability to environmental impacts over the past decade now documents dual influences of both biophysical factors and social conditions that make communities vulnerable. However, few studies have examined how these broader understandings of environmental threats may be perceived among different segments of the public – the focus of the present research.

Group differences in the experience of environmental risks, or awareness that these disparities exist, may hold implications for how different groups conceptualize environmental issues. Although theories from the environmental justice literature have discussed this possibility [1,^22^,44], limited empirical research in the environmental psychology literature speaks to this possibility directly. Whereas environmental science scholarship and advocacy has traditionally focused on ecocentric environmental issues (e.g., industrial pollution, flooding, drought), reflecting biophysical hazards, environmental justice broadens this focus to consider social conditions that magnify human harm (e.g., racism, poverty), as well as the disparate impact of environmental harms across groups [25,26]. Indeed, communities of color have long protested issues such as inadequate sanitation, lead poisoning in urban areas, and asbestos in schools and work at the local level, and placed these on the agenda of the Civil Rights Movement [32]. According to Taylor [44], due to their cultural roots, mainstream environmentalists appealing to the White middle-class tend to associate images of wilderness and wildlife protection with romanticized 19th century experiences; in contrast, environmental justice advocates may evoke cultural images of racism, land appropriation, and community destruction associated with the same era.

Because previous research documented that White and wealthier Americans had different exposure to environmental hazards and political experiences with environmental issues than their racial and ethnic minority and lower socioeconomic status peers, Song and colleagues [40] examined whether those differences have implications for how different groups of Americans construe environmental issues—that is, whether they have different definitions of what “counts” as an environmental issue. They conducted a national survey to explore that research question in a quantitative manner. That survey was developed not only in light of these previous findings in the literature, but also due to a qualitative study conducted with residents in a low-income racial and ethnic minority community in a major US city. The goal of this article is to describe that qualitative study, and its implications for understanding the quantitative findings reported in Song and colleagues [40], as well as the broader implications of incorporating qualitative approaches for improving quantitative inferences in environmental psychology research.

## Method

Our investigation of racial and economic group differences in environmental issue conceptualization began, admittedly, by accident. After discovering that racial and ethnic minority and low-income Americans express the highest levels of environmental concern in national opinion polling but are stereotyped (even among themselves) as having the lowest levels of concern [31], we wondered whether this misperception could be addressed with a social norm intervention [43], and whether correcting the misperception would have downstream consequences for environmental engagement. To develop our normative intervention, we decided to conduct focus group research with Latina/o community group members to gather information that could be used to create motivational intervention messages [7]. We chose to focus on Latina/o participants because in our previous research on environmental concern, that was the group whose environmental concerns were misperceived the most. That is, although other groups also face environmental hazards and are misperceived as not caring about the environment, our previous nationally representative studies documented that Latina/o Americans are the group of Americans who are most concerned about the environment, but are perceived as being the least concerned [31]. This misperception occurs even among Latina/o participants—Latina/o participants report high levels of concern about the environment, but when asked how concerned other Latina/o people are about the environment, they perceive fellow Latina/os as significantly less concerned than the self-report data suggest. Correcting such misperceptions is important given that misperceiving ingroup norms may deter groups from partaking in collective action (see [31]).

Given these previous findings, we partnered with the Environmental Defense Fund to leverage their network of Latina/o community organizations to recruit participants for our research study. Through this partnership we decided to recruit participants in San Antonio, Texas. San Antonio is a majority Latina/o city that is home to one of the largest Hispanic populations in the U.S., which made it an ideal location to recruit a sufficiently sized sample to gather information to create the messages we hoped to generate and test. Thus, the primary goal of the focus group study was to elicit leading environmental and sustainability issues that could be used in the creation of normative messages (see [7]). As described in the remainder of this article however, once we conducted the focus groups, the goal of the research changed based on what we learned, as sometimes happens when researchers step outside of the laboratory and interact with people rather than just the datapoints in our spreadsheets [5,^11^,21]. These focus group findings introduced a different set of questions, which led us to first investigate potential group differences in environmental issue conceptualization and their antecedents and consequences, using a semi-structured interview protocol adapted from Krueger and Casey [16] – a comprehensive and practical guidebook for conducting focus groups for applied research purposes.

### Interview procedure

Our team worked with community partners to recruit 24 representatives from 16 Hispanic and Latino community organizations working in and around San Antonio. The focus groups were hosted in a community center in a low-income neighborhood on the city's Westside (zip code 78207). The organizations themselves were diverse in mission and scope, and included those working on: voter registration, business development, culture and arts, and promoting access to healthy food. Invitees were originally contacted by a member of our research team and informed about our plan to hold small, informal focus groups focused on the following questions: “What are the top issues facing your community?” “What environmental changes would you like to see?” “Do your friends and family care about the environment?” These questions were designed to inductively illuminate specific topics that participants associated with environmental degradation in their communities. Invitees were further informed that the output from these focus groups would shape the next steps of our research, including our plan to investigate how different ways of communicating about the environmental beliefs and concerns of different communities would affect people and motivate them to take environmental action.

Participants attended one of three semi-structured focus groups discussions, each of which involved between six and nine participants, in addition to the moderator. For observation and data-recording purposes, three other members of the research team were present in the room but did not sit at the main table or participate in the discussion – they took notes as the conversations progressed. One of the three^1^ focus groups was audio-taped for transcription, to compare to written notes of the research team. Upon arrival, participants provided informed consent and were reminded that the purpose of the discussions was for our research team to learn about “issues facing your community in San Antonio.” Moreover, participants were informed that the purpose was not to establish consensus and that there were no right or wrong answers, but rather, our goal was to have them generate as broad a list of issue priorities as possible.

The moderator then proceeded to walk the group through a semi-structured interview protocol (adapted from [16]), which began with asking participants to generate “three issues that seem most important” to the Latina/o community in San Antonio. After about a 1-minute pause to allow participants to privately reflect, the moderator invited each participant to share the three issues they identified, which were recorded as a list on a large notepad by another member of our team (the notetaker). As the moderator went from participant to participant and novel issues were shared, they were added to the growing list which was visible to all participants. This was intended to encourage discussion about the ways in which different issues were the same, related but not the same, or entirely different. Throughout this reporting process, the moderator and notetaker were mainly passive observers; they did, however, prompt participants to clarify their comments if they were unclear in and of themselves, or in their relationship to other issues that had already been recorded.

After hearing from everyone, the moderator proceeded to the second prompt, which asked participants to generate, specifically, the most important *environmental* issues facing their communities. Once again, the protocol asked the moderator to pause to allow time for each participant to identify issues and reflect, although in some cases, the discussion was proceeding organically by this point and participants simply volunteered issues as they occurred to them. As before, the notetaker listed novel issues on the large, visible sheet of paper.

Following the discussion of important issues in general and of important environmental issues specifically, the protocol called for the moderator to prompt participants to name any issues that had not yet been named, and as a final question, to share “one thing” they would tell the mayor of San Antonio, if they were given the opportunity. This closing question was intended to encourage participants to think concretely about community problems and their potential solutions—to mitigate the possibility that framing questions in terms of “issues” might have invited responses that were too abstract for the purposes of our research questions; it also allowed participants to highlight which issues they thought were the most important. Finally, participants were thanked, provided with a paper survey to report any additional comments, and given a debriefing form with information to contact us if there was anything else they wished to discuss. Altogether, each focus group session lasted about 1.5 hours.

### Coding and analysis procedure

Following the focus group discussions, the research team that was on site for data collection (all senior members of the research team) returned to our hotels to transcribe our handwritten notes and upload our typed notes to our secure Server. After we returned to campus, the graduate student members of our research team took the large notepad posters which contained participant responses from each focus group and transcribed the issues that were discussed in each focus group. They also transcribed the audio file from the one group for which we had an audio recording, but we decided not to include that in our coding as that would have introduced an imbalance in the amount of information available for each group (i.e., one focus group would have had more information, and of a different source, than the other focus groups).

After all files were transcribed, one of the graduate student members of our research team went through all transcription files to extract key themes from the focus groups. She began by conducting a frequency count of issue mentions in each group, and then identified the convergence of issues across groups. Specifically, groups varied in the number and type of issues mentioned: the first group generated 34 different issues, the second group generated 22 different issues, and the third group generated 16 different issues. We discussed these issues as a larger research team to determine whether there was convergence across the groups that could be generative. Although issues varied across groups as a function of the particular people who were present, there were some consistent issues that were raised in every group and emerged as common themes. These themes informed the development of the research questions and survey described by Song and colleagues [40].

## Results

Of the 34 issues mentioned in the first focus group the issues that came up most frequently were: poverty, low wages, and employment (6 unique mentions), educational opportunities (5 unique mentions), disparities and economic inequality (3 unique mentions), and health (3 unique mentions); other issues only received one unique mention from respondents. Of the 22 issues brought up in the second focus group, the issues that came up most frequently were: education (5 unique mentions), economic inequality (3 unique mentions), access to nutritious food (3 unique mentions), walkability in the city (3 unique mentions), issues with mass transit (3 unique mentions), and people not voting (3 unique mentions); other issues received only one unique mention from respondents. Of the 16 issues mentioned in the third focus group, the issues that came up most frequently were: air quality (4 unique mentions), flood and drainage issues in the city (3 unique mentions), educational opportunities (3 unique mentions), and poverty (3 unique mentions). After seeing both *what* participants brought up consistently across groups as well as *how* they discussed these issues, it became clear to us that our participants’ environmental concerns could be synthesized into two emergent themes.

One emergent theme can be characterized as *environmental issues are social issues.* When participants were asked to identify the most important environmental issues facing Latina/os in San Antonio, they were quick to highlight the links between what are typically viewed as environmental issues and the social issues that had been previously discussed. For example, issues like air pollution and lack of green spaces were explicitly discussed as intertwined with a host of social issues, including racism, economic inequality, and health problems (notably, obesity). One notable example concerns discarded drug needles on lawns and walkways in poorer neighborhoods, which multiple participants cited as a leading environmental issue (as a form of litter, and because it discourages residents from safely accessing and enjoying outdoor spaces) that is inextricable from inequities in education, employment, and health care access in one of the poorest zip codes in the U.S. A related example concerns the lack and poor maintenance of sidewalks in the city's lower-income neighborhoods, which participants linked to multiple effects—including air pollution due to an increased reliance on cars, restricted access to (already limited) green space, and limited opportunity for physical exercise.

Another emergent theme can be characterized as *environmental issues are local issues.* When asked to name leading environmental issues facing their communities, focus group participants tended not to mention issues playing out at the national or global scale (e.g., climate change, fracking), but rather, they tended to name issues that they perceived as affecting certain neighborhoods in San Antonio more than others, as demonstrated by the drug litter example above. Other examples endorsed by multiple participants included localized flooding resulting from inadequate storm-water infrastructure in low-income neighborhoods of mostly Latina/o residents and differential exposure to harmful soil and air pollution originating from a nearby military base. Indeed, participants explicitly acknowledged that members of their communities might be primarily concerned about localized issues that they encounter on a daily basis (“[It's] not like global warming,” one participant noted when describing the drug litter issue), while members of more-affluent communities elsewhere in San Antonio might be more concerned about broader-scale environmental issues (“someone from the North side isn't going to talk about drug needles,” one participant explained, to which another replied, “somebody from the North side would probably say ‘fracking’”).

## Discussion

### Significance and applications

Prior to conducting this focus group study with members of Latina/o community organizations in San Antonio, we expected that discussions would revolve around the impacts of environmental issues that are discussed more frequently in the environmental psychology literature—issues such as climate change, air quality, and water quality. Although those issues did surface, the discussions were dominated by issues that have received greater attention in the environmental justice and environmental racism literatures—including issues that represent social determinants and consequences of environmental risks, with lack of education, poverty, built environments (i.e., lack of sidewalks) and obesity being notable examples. Moreover, much of the discussion focused on drawing out the interconnections that participants saw between what we ended up terming *human-oriented* issues (e.g., poverty, lack of access to grocery stores) rather than more *eco-oriented* issues (e.g., climate change, industrial pollution) that are more frequently discussed in the environmental psychology literature [40]. For example, in our participants’ minds, inadequate sidewalks were inextricably linked to poor air quality because the lack of sidewalks (and other reliable public transit) forced residents to drive more frequently, and the cars that people in their neighborhoods could typically afford tended to be older and more polluting than those in wealthier San Antonio neighborhoods.

Conducting this focus group study made salient and explicit a phenomenon that environmental justice and environmental racism scholars have written about, but has received limited empirical attention, to-date, in environmental psychology: that group-based differences in vulnerabilities to environmental hazards that have emerged due to structural inequities [2,^25^,^26^,45] may lead members of different groups to different conceptions of what “counts” as an environmental issue [40]. The principal finding from Song and colleagues [40]—that issues including poverty, drug abuse, and racism are more likely to be counted as environmental issues by non-White and lower-income survey respondents, compared to their White and higher-income counterparts—is consistent not only with environmental justice perspectives, but also research in psychology on how differences in social structure shape the lenses through which people interpret the world around them [30]. Had we not held those initial focus groups, we likely would have surveyed our respondents about a narrower set of issues, or have been less equipped to make sense of our quantitative results. Understanding how the qualitative patterns in empirical data emerge from models of psychological processes is essential for making inferences about quantitative indicators [27]. In this way, our team came to more fully appreciate the value of combining qualitative examinations with quantitative approaches, and in particular, the importance of linking environmental psychology, environmental justice, and environmental racism perspectives when seeking to deepen our understanding of whether and how different groups may think differently about environmental issues [3]. We limit our ability to generate useful explanations and predictions when we employ one approach without the other.

### Limitations and Constraints on Generalizability

Although informative for improving the quantitative study reported by Song and colleagues [40], and more generally for thinking about quantitative inferences in environmental psychology, the current study has some important limitations that must be considered when interpreting the findings and their generalizability [39]. We recruited a sample of Latina/o individuals from San Antonio, Texas to participate in our study. As noted earlier, this decision was driven by findings from previous research documenting misperceptions of the environmental concerns of this group among the US public [31]. San Antonio is a city wherein the vast majority of residents are Latina/o; in other words, it is a “majority-minority” city. The bulk of environmental justice and environmental racism literatures sample participants from low SES minority populations in settings in which they are minorities within a majority race population. While we do not yet know how these contextual differences might affect the results found in the current paper, a growing body of meta-scientific research suggests that there is often substantial variability in social scientific research by context (e.g., [15]), and thus future research should explore this more systematically due to its implications for both theory advancement and practical application [9,39].

### Final Note

Despite the limitations of the current findings, we believe there are important theoretical, practical, and ethical gains associated with combining qualitative and quantitative methods in (environmental) psychology. We discussed the theoretical and practical elements in the previous sections, and want to end by discussing the ethical argument—the argument about the implications of our methodological practices for our research's influence in society [4,18]. Over the past few years, as occurs every few decades [4], professional psychological science organizations have been encouraging psychologists to “give psychology away” in hopes that psychologists can have similar influences on public policy as our colleagues in economics [46]. If research in environmental psychology is to inform social policies about environmental issues, then it ought to not only include broad representation of groups that are disproportionately affected by environmental hazards [18], it also needs to reflect deeper perspectives of people from those groups—insights gained by combining quantitative techniques with qualitative techniques.

## Footnotes

1 One group did not consent to being recorded; in another group the moderator forgot to press record at the beginning of the session.
